# Solar radio emission as a disturbance of radiomobile networks

**DOI:** 10.1038/s41598-022-13358-z

**Published:** 2022-06-04

**Authors:** Giuliano Muratore, Teresa Giannini, Davide Micheli

**Affiliations:** 1 Telecom Italia, Rome, Italy; 2 Istituto Nazionale Di Astrofisica (INAF), Rome, Italy

**Keywords:** Solar physics, Solar physics

## Abstract

This paper analyses the effects of solar radio emissions in the radiomobile context, for the first time leveraging massive European Telecommunications Standards Institute (ETSI) 3rd Generation Partnership Program (3GPP) Minimization of Drive Test (MDT) radio measures produced by 4G LTE (Long Term Evolution) terminals and by 4G LTE Base Station cells. A method to study solar noise effects starting from radiomobile 3GPP standard MDT measures is illustrated and correlated with the excellent 10.7 cm (2800 MHz) indicator of solar activity (National Research Council Canada). The effects of solar disturbance on the LTE radio access network for mobile services are analysed, and possible countermeasures are presented from the perspective of radiomobile network evolution to 5G and 6G.

## Introduction

Solar flares are a well-known phenomenon that creates a sudden release of a great amount of energy out of the Sun and within Sun high magnetic field regions, called solar active regions^1^. During flares, energy confined by magnetic fields is rapidly converted into kinetic, thermal and mechanical energies, capable of locally heating plasma to several million degrees and accelerating ambient particles to high energies. All these phenomena also produce electromagnetic field emission with radio waves covering an extremely wide frequency range, starting from tens of kHz up to a few hundred GHz^2^. An approximately 11-year time period, characterized by a Sun’s magnetic pole flip, alternates the time periods of the quiet Sun with a time period of intense solar activity mainly characterized by events such as solar flares and coronal mass injection (CME) with high energy flux particles and radiation emissions. The last solar cycle (24°) was a feeble cycle, peaking at 114 sunspots (the average was 179), and communication services were rarely badly affected by solar radio emissions^3^. The recently initiated 25° solar cycle shows some meaningful flare events, such as the 3rd of July 2021 classified of magnitude X1.5 (Geostationary Operational Environment Satellites classification in a range from A, B, C, M to X, according to the peak flux in watts per square metre of X-rays with wavelengths 100 to 800 pm).

This article, for the first time, studies the solar radio disturbance of LTE services by means of innovative 3GPP^4^ MDT (Minimization of Drive Test) standard radio measures and the richness of the R language environment for data elaborations^5^. The MDT^6–13^ standard allows the mobile network operator (MNO) to collect georeferenced radio measurements anonymously and free of charge reported by mobile phones to the network, measurements including radio received base station (BS) cell power levels, interference levels and quality of radio links at the frequencies of MNO serving BS cells. In this way, a huge amount of periodic radio measurements related to the territory around BS cells can be analysed with data science methods to extract statistical information on reference signal received power (RSRP) and interference signal to interference ratio (SIR) fluctuation.

The research on the discovered solar disturbance of LTE services is organized in four sections.

The *Results* section highlights the main evidence of the study.

The section Cellular network radio measures and solar noise describes how to leverage 3GPP MDT standard radio measures to detect solar noise disturbance and how the estimated solar noise disturbance is correlated to official Penticton radio-telescope measures of solar noise (provided as a service by the National Research Council of Canada for many scientific and commercial activities at both industrial organizations and government agencies around the world).

The section *Observations* focuses on July the 3rd flare (X1.5 class) and compares solar effects on network behaviour with the immediately preceding July the 2nd day, when Sun was still quiet.

The section *Radiomobile data analysis* shows the effects of the 3rd July flare on a 4G LTE RAN, detailing the reasons behind quality drops and possible countermeasures, specifically for the RAN evolution toward 5G and 6G.

## Results

An analysis of solar effects on radiomobile 4G LTE networks was conducted by means of massive 3GPP MDT radio measures produced by millions of phones connected to MNO LTE BS cells available in northeast regions of Italy. A good correlation was found between an index extracted by radiomobile measures and the F10.7 indicator of solar activity.

The radiomobile index was analysed for the sunlight hours of July 3rd, day with a class X1.5 flare, comparing effects with days of quiet Sun.

Effects on mobile communication quality of service were identified in relation to radiomobile BS cell horizontal orientation (BS cell azimuth) toward the Sun, whereas effects were weakly influenced by BS cell vertical orientation (antenna elevation angle, usually called BS cell antenna tilt), thus indicating a way for countermeasures capable of limiting solar disturbance of radiomobile networks, also from the perspective of 5G and 6G RAN evolutions towards self-organizing networks (RAN that automatically plan, configure, manage and optimize themselves).

The solar irradiance in the microwave region, specifically in bands at approximately 3 GHz, can deeply vary from the situation of a quiet sun to the situation of an active sun. During higher peaks of solar flux (e.g., 1000 or even 10,000 solar flux units, or briefly s.f.u., corresponding to 10^−22^ W m^−2^ Hz^−1^) at approximately 3 GHz, the gap between the signal power received by mobile phones and solar noise power can become narrower, so the communication quality of service can be even more deeply affected with respect to the situation discussed in this article.

Solar effects are even more probable in radiomobile higher bands (24 GHz–40 GHz) allocated for 5G, with solar irradiance being stronger (with lower magnitude bursts) and less dependent on the solar quiet or active period. In this 5G solar scenario, the flexibility offered by electronic beam orientation can be leveraged to reduce radio link quality degradation, maintaining the beam orientation toward the user position as much as possible and preferring beam orientations not aligned with the Sun azimuth.

Future analysis, making use of the first available 5G MDT measures in 3.7 GHz band, is expected to improve the knowledge about solar effects on radiomobile networks, considering the relevance of 5G (and future 6G) connections for many innovative services and industrial applications.

The presented analysis is based on fully standard radiomobile MDT measures collected by 8800 cells in territory. Such measures are potentially available H24 in all running radiomobile networks worldwide (4G, 5G, etc.). In this way, the extension of the proposed approach to a planetary scale (many radiomobile networks located on different continents) theoretically enables the analysis of whatever source of “noise” comes from space within the radiomobile frequencies, ideally creating a “virtual wordwide radiotelescope” that, leveraging the Earth curvature, can also estimate the extraterrestrial source elevation.

## Discussion

### Cellular networks radio measures and solar noise

All cellular networks continuously monitor the radio frequencies allocated for the communication services offered to the customers and constantly receive, from each user equipment (UE), such as smartphone devices, the signal level of the cells around it and the related channel quality indicators.

UEs to periodically send a large set of measurements from Layer 2—MAC (3GPP TS 36.321),—and Layer 3—Radio Resource Control (3GPP TS 36.331) and, with MDT, UEs share their measurements periodically with the network (when a global positioning system (GPS) receiver is enabled, UE measurements are also geolocated).

This standard mechanism creates billions of daily measures describing radioelectric evolution around each cell, essential information to optimize frequency band usage and fight the impact of radio interference and (inevitably) environmental electromagnetic noise such as thermal noise, both coming from Earth and sky.

Big Data analysis, a quite recent but now consolidated industry trend, enables new investigation directions capable to leverage massive radio data including also less frequent events, like (but not only) solar activity disturbances.

The study of Solar effects at 2695 MHz on 4G LTE systems by means of MDT paves the way for studying effects for the new 5G radio access adopting sub millimeter band below 6 GHz and, in the near future, millimeter waveband above 25 GHz.

Sun could be more disturbing at 5G and 6G higher frequencies and, in the meantime, radiomobile services are extending their reach to include low latency mission critical uses (e.g. in contexts like: autonomous driving, remote surgery, energy and water active grids, etc.).

RAN LTE installations are typically used in a three-sector configuration^14^ to optimize capacity and radio coverage, combining three 120° sectors (e.g., half power at + /− 60°), all the (360°) territory around. Cells are characterized by their physical position, by the frequency band allocated to communicate with mobile terminals, by the horizontal orientation (azimuth, maximum antenna gain direction) and by the vertical orientation, technically also said the tilt. Typically, BS cell antennas use a negative tilt (i.e., pointing down toward streets and buildings where people live) or zero tilt (i.e., pointing toward the horizon). Therefore, in general, cells do not point toward the sky, and antenna gain decreases more sharply in the vertical direction (e.g., half power at + /− 10° degrees). Practically speaking, solar radio emissions could directly inject noise in radiomobile antennas, even when the cell azimuth matches the Sun azimuth, only during sunrise or sunset, when tilt is somehow aligned to the (zero) solar elevation in the sky.

Nevertheless, we cannot forget that solar radio waves coming from the sky and impacting ground (or roofs, buildings, roads, etc.) are subject to reflections and more in general scattering. Indeed, by considering the typical decimetre wavelength of many mobile bands worldwide, solar radio waves could also be bent by diffraction from edges all along the Earth’s surface or by diffraction from human infrastructures on the Earth’s surface (e.g., buildings, walls, cars). Electromagnetic scattering, the phenomenon that makes it possible to serve a huge mass of mobile terminals without the need for direct antenna visibility, weaks the role of the BS cell antenna tilt as a protection from Sun radio disturbance, allowing injection in BS cell antennas part of the solar radio disturbance when tilt is not aligned to the Sun elevation. The only practical protection from solar radio disturbance remains the BS cell antenna horizontal orientation (cell azimuth).

**Figure 1:**
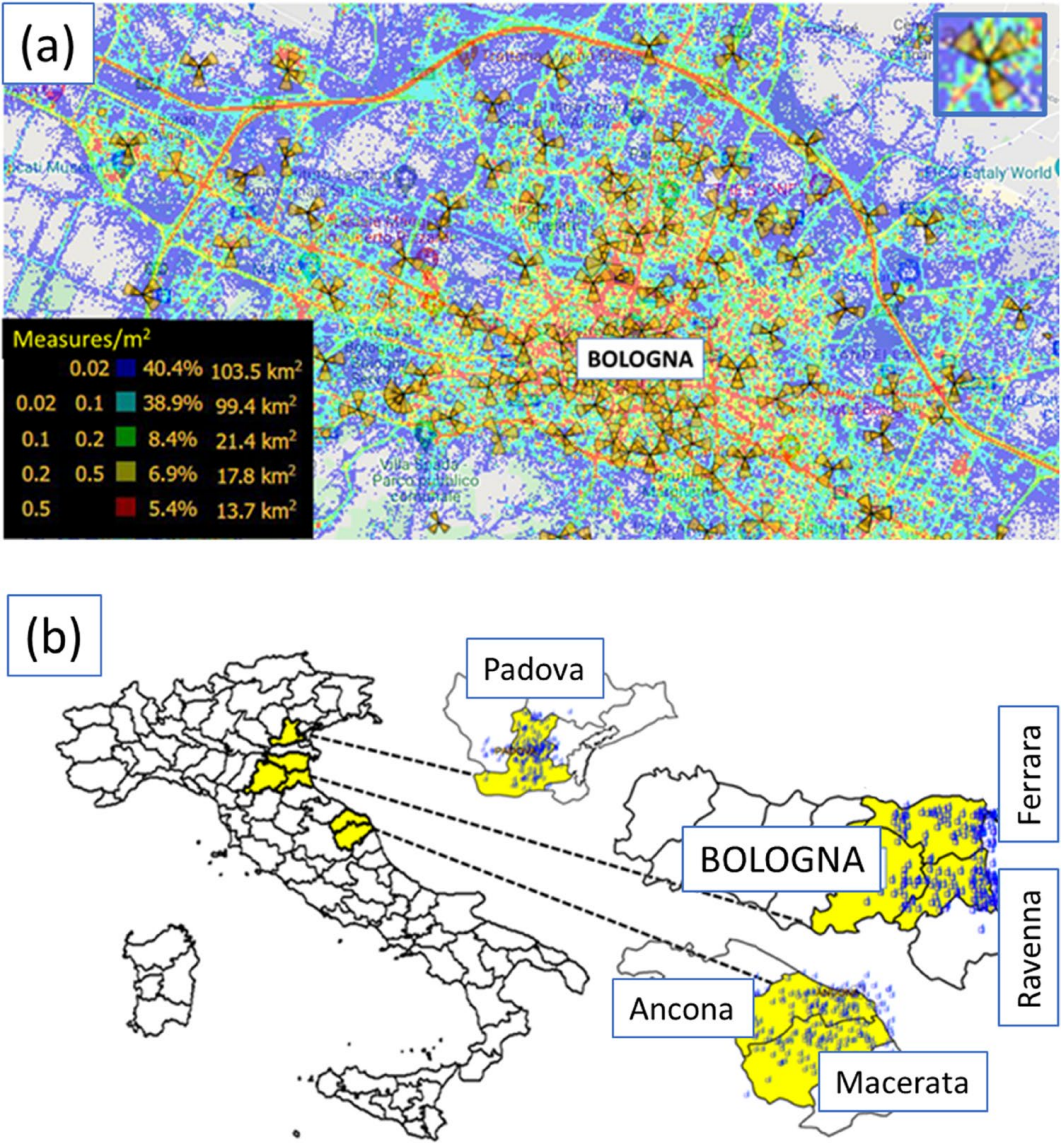
(**a**) A view of MDT sample density [measures/m^2^] in Bologna City. Cells are visible as triangular oriented sectors. Points for the geographical mapping representation are pixels having a 1 m side length. (**b**) The six different Italian Nord-Est areas (in yellow) involved in the study collectively cover 15.051 km^2^.

To isolate Sun noise disturbance on radiomobile RAN from other interferences, two criteria are considered. The first criterion takes advantage of the abundance of radiomobile cells insisting on a sufficiently wide geographical area (see Fig. 1). Indeed, a single cell can be affected by local noise around the cell, whereas thousands of cells spread over a wide area allows mitigation of local interference phenomena.

**Figure 2:**
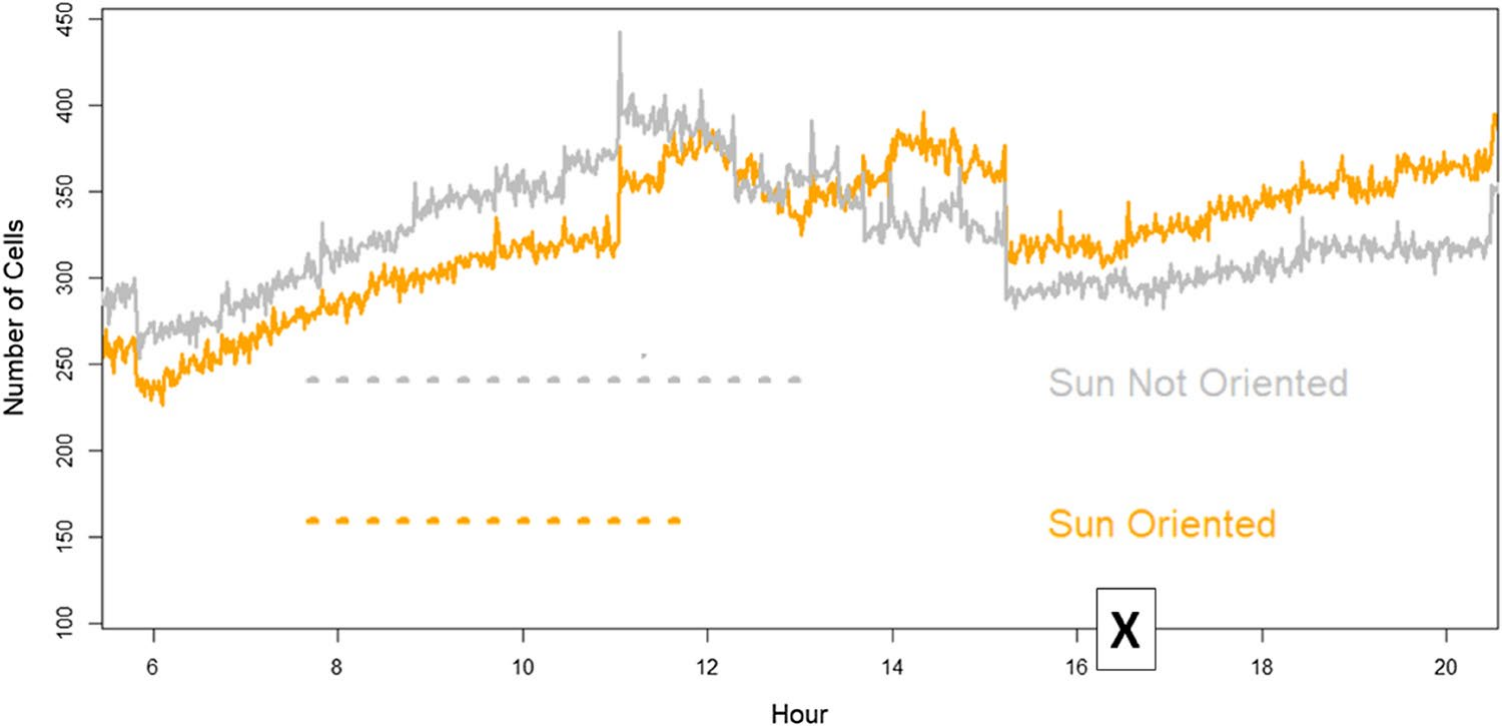
An example of the number cell trend during July 3rd in one (Bologna area, 3702 km^2^) of six areas involved in the study. “X” indicates the flare peak in X-ray emission (local Italian time, UT + 2H).

The second criterion exploits the abundance of different cell orientations (azimuth), permitting always (at each time of the day) to select a specific subgroup of cells that, in that moment, result (horizontally) oriented toward Sun azimuth and another subgroup of cells oriented in the opposite way. Using this approach, Sun noise RF power can be followed throughout the day (see Fig. 2), selecting, at each moment, the appropriate subgroup of cells and analysing the behaviour differences between the two subgroups. The Sun-exposed subgroup is characterized by the fact that the maximum antenna gain is in the direction of the Sun. In contrast, the other subgroup of cells exposes the minimum antenna gain in the direction of the sun. Effects depending specifically on cell orientation toward the Sun can emerge from this dynamic division of cells in the two groups, analysing the radio measures collectively produced by the single group of cells and comparing the two results.

**Figure 3:**
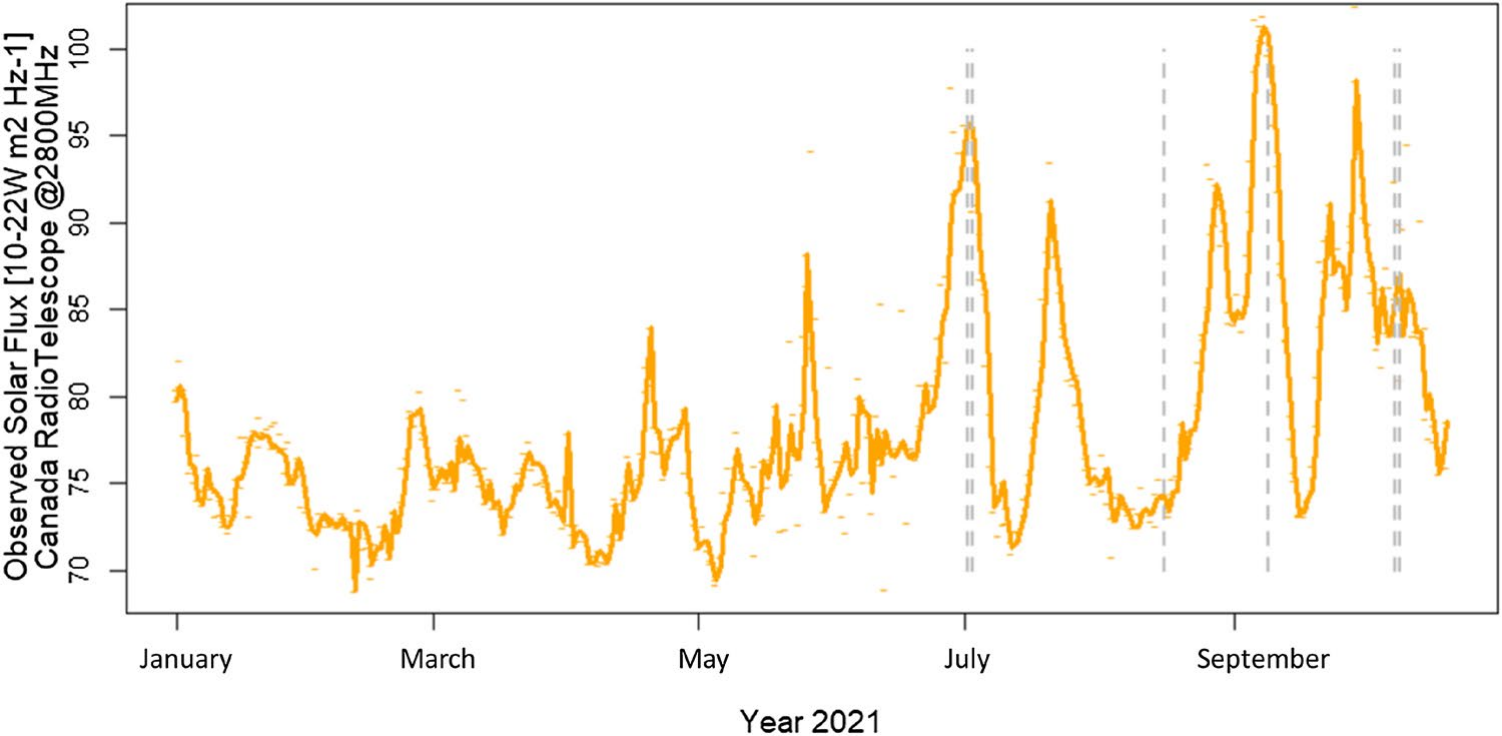
The Penticton F10.7 Index in 2021, and the 6 days (gray vertical lines) of July 2nd and 3rd, August 16th, September 9th, October 8th and 9th used to compare F10.7 Index with the disturbance derived from radiomobile MDT measures at 2.6 GHz.

In terms of frequency, the choice was to analyse the 2.6 GHz band (2500–2690 MHz), which was allocated by the World Radiocommunication Conference (WRC) in 2000 for terrestrial mobile communications services. The analysis of the 2.6 GHz band presents two advantages. First solar radio emission is typically stronger in 2.6 GHz band, with respect to the other lower frequencies currently used for radiomobile communications. Second, the 2.6 GHz band is currently the closest LTE band to the frequencies (in the 100 MHz range from 2.75 GHz to 2.85 GHz) that the Penticton Radio Observatory consistently monitors. Solar radio flux at 2.8 GHz is also referred as the F10.7 index. The index (10.7 being the wavelength in centimeters) is an excellent indicator of solar activity since 1947, easily and reliably measured on a day-to-day basis (see Fig. 3) in all types of weather and well correlated with sunspot number^15,16^.

The Penticton F10.7 index, expressed in s.f.u., is composed of three daily solar flux measures, each representing a one-hour average. The time zone difference (9 h) between Canada (Penticton) and Italy guarantees Sun observation contemporaneity (during summer) only for the first daily Canadian measure at 10:00 a.m. in Penticton (17:00 UT), an hour that corresponds to 19:00 in Italy.

**Figure 4:**
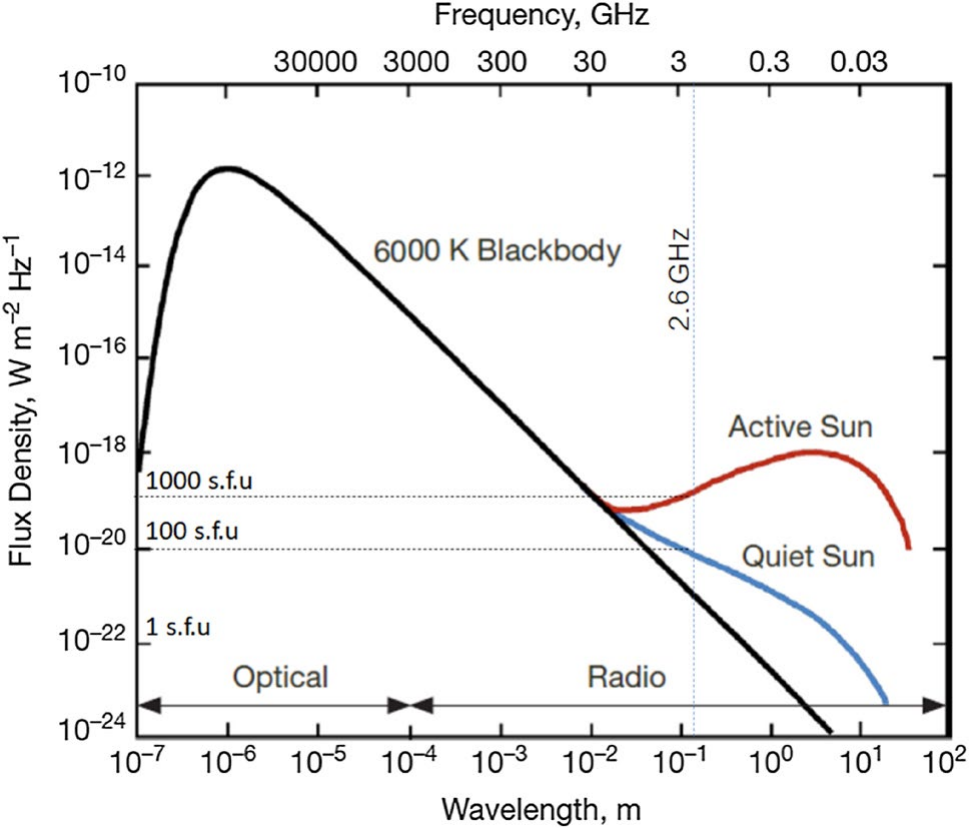
Solar radiation spectrum at optical and radio frequencies extracted from Christian Ho, Stephen Slobin, Anil Kantak, and Sami Asmar^17^. At wavelengths greater than 1 cm, the active sun (red line) and quiet sun (blue line) deeply differ in terms of radiance, not following blackbody emittance at 6000 K (black line). The specific situation at 2.6 GHz is added with the dotted lines, showing one order of magnitude s.f.u. variation (approximately in the range 100–1000 s.f.u).

A general characterization of solar noise at 2.6 GHz is visible in Fig. 4, showing the great difference existing, at those frequencies, between quiet Sun and active Sun conditions.

To study Sun influence on radiomobile services, a relevant radio measure is the Up Link Signal Interference Noise Ratio (UL_SINR), i.e., the ratio (typically expressed in dB) between the power a BS cell receives when a signal is sent (Up Link direction) by a mobile terminal, and the interferences and noises the same cell detects during communication with the mobile terminal on the same Resource Blocks (RB being the smallest unit of radio resources that can be allocated to a user, 180 kHz wide frequency).

Leveraging UL_SINR measures on UpLink RBs allocated at 2.6 GHz, we defined a different index, the “UL_SINR Index”, to describe the solar activity influence on radiomobiles. The UL_SINR index is built by taking the difference between the median of the UL_SINR distribution measures related to cells that result directed toward the Sun azimuth (with a ± 60° tolerance) and the median of the UL_SINR distribution measures related to cells that result directed in the opposite way (again with a ± 60° tolerance). By averaging these differences between UL_SINR distribution medians over one hour and changing the group of cells according to the path of the Sun throughout the day, we obtain a value, the UL_SINR Index (expressed in dB), that can be compared with the corresponding F10.7 Index (of the same hour). The idea behind this index is to maximize the Sun noise detectability with respect to mobile phone interference, typically randomly spread around the RBs assigned by the serving cell.

Considering that radiomobile cells are distributed throughout the territory in a way that tends to maximize radio coverage and mobile phone traffic and at the same time points to minimize reciprocal cell interference, there is no reason to have a UL_SINR distribution median that depends on a specific cell orientation direction when we consider a high number of cells and measures to populate those distributions. Therefore, the general expectation is that the UL_SINR Index is close to zero. When a difference exists, and this difference is negative, it means that it operates a cause that makes the UL_SINR lower for the Sun oriented cells and higher for the cells oriented in the opposite way. In other terms the anti-correlation between Penticton F10.7 index and the UL_SINR index show the existence of an influence of the Sun on the radiomobile uplink channel quality, with the SINR being one of the indicators of good or bad communication on the radio (uplink) channel.

## Observations

In Table 1 the values of the Penticton F10.7 index is reported (2nd column) for 6 different days (1st column). Days are selected to obtain a mix of solar conditions at 2.8 GHz (higher s.f.u, medium s.f.u, lower s.f.u) during the observation period. The corresponding (same day and same hour in the first four cases, same day but contiguous hours in the last two cases) UL_SINR Index (3rd column) is −82% (anti)correlated with F10.7 Index, showing that the UL_SINR Index is a good indicator (correlation test p-value 0.04) of solar noise influence on the radiomobile context.

**Table 1:** Comparison between Canadian F10.7 Index (2.8 GHz Penticton Radio-Telescope) and UL_SINR Index (derived from MDT radiomobile measures in 2.6 GHz band).

Day year 2021	CANADA—F10.7 Index s.f.u.@2.8 GHz (16:30–17:30 UT)	ITALY—UL_SINR Index Delta (dB) @2.5 GHz (16:30–17:30 UT)	Note
July 2nd	95.7	−0.124233	
July 3rd	95.5	−0.5545361	X1.5 class flare peak at 14:29 UT, two hours before Canadian F10.7 Index calculation. The corresponding UL_SINR Index (−0.5545361) was calculated averaging 466,369 MDT measures reported by 783 different LTE cells
August 16th	74.3	0.1835652	
September 9th	100.7	−0.3704581	
October 8th	85.7	0.1198423	Due to sunset time in October in Italy, the October UL_SINR Index refer to the hour 15:30–16:30 UT (one hour before F10.7 Index calculation)
October 9th	87.1	−0.04971983	

Once established, the UL_SINR Index along the 16:30–17:30 UT hour approximates the s.f.u monitored by Penticton F10.7 index, UL_SINR Index can also be used in relation to hours when there is no corresponding Penticton F10.7 index measures available. Specifically, in the following paragraph, all the solar hours of the most interesting day (July 3rd, showing X1.5 class flares at 14:29 UT) are investigated, focusing on the effects on radiomobile connections.

**Figure 5:**
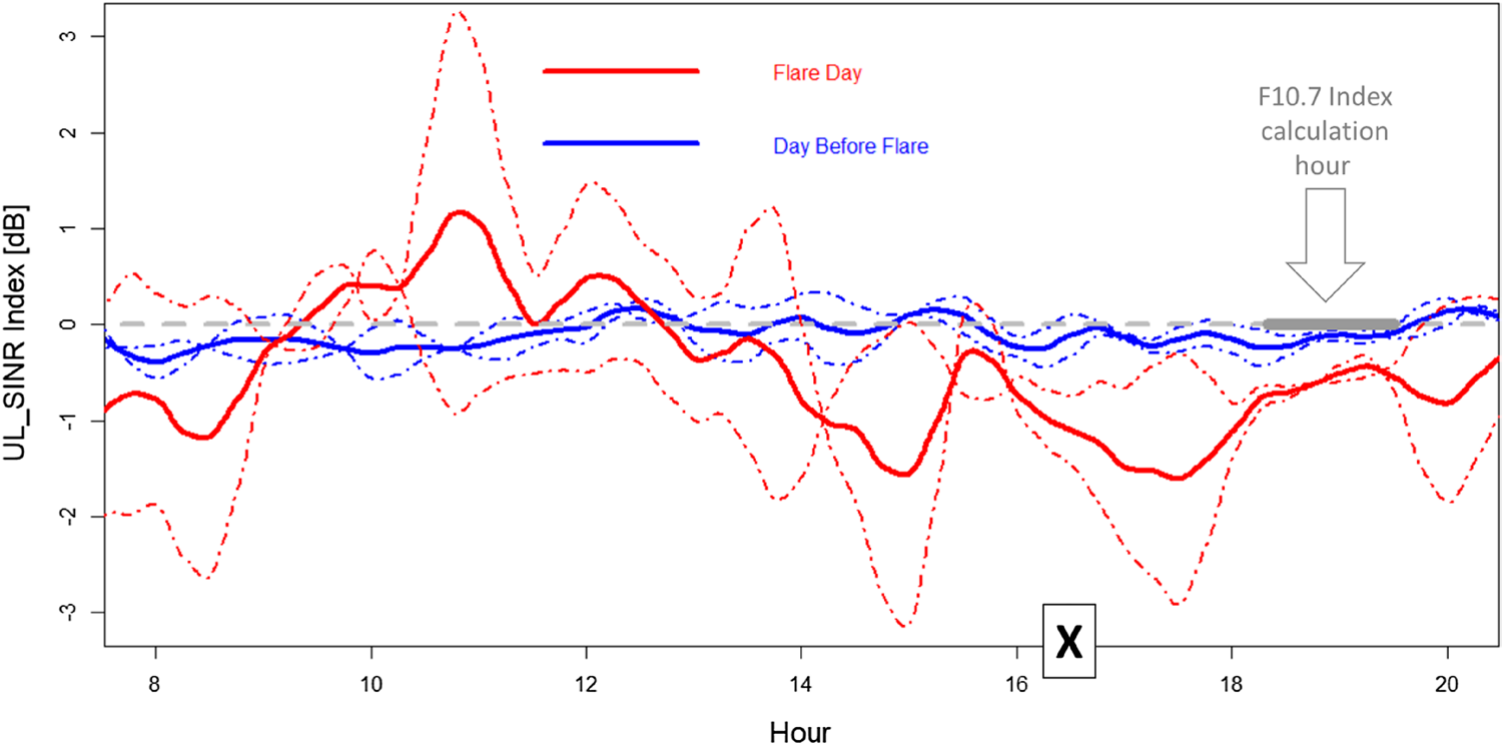
Full day comparison between the UL_SINR Index (derived from MDT radiomobile measures in 2.6 GHz band) during July 3rd (red line) and during the day before (blue line), with more quiet Sun. The dotted and dashed lines indicate the standard deviation (in blue for July 2nd, red for July 3rd) of the two UL_SINR distributions (whose medians are the two compared UL_SINR indexes) describing the UL_SINR difference between Sun-oriented and non-Sun-oriented cells. “X” indicates the flare peak in X-ray emission (local Italian time, UT + 2H). The thick gray segment (see gray arrow), along the dotted gray lines (indicating UL_SINR Index zero), shows the hour of the, a 60-min period of minor solar emission at 2.8 GHz and lower solar emission difference between the two consecutive days.

The UL_SINR index calculated, minute by minute, during the sunlight hours of July 3, 2021 (Sun flare peak at 16:29 local Italian time) and along the previous day (Sun still quiet) is reported (thick lines) in Fig. 5, showing a clearer separation between the two day UL_SINR distributions (whose medians are the two compared UL_SINR indexes) in the period immediately before and after the flare.

The 2nd of July day (quiet Sun) shows a quiet UL_SINR index trend, almost zero, meaning that the signal-to-noise ratio related to Sun-exposed cells remained always similar to the signal-to-noise ratio of the cells oriented in the opposite way. In contrast, specifically during July 3rd afternoon, the signal-to-noise ratio of the cells oriented toward the Sun became lower than the UL_SINR of the cells oriented in the opposite way, so moving the UL_SINR Index deeply down on two occasions (before and after the X-ray Sun peak).

It is noticeable that the Pentincton F10.7 index registers solar noise at fixed hours, and it registered the solar noise at 2.8 GHz between 18:30 and 19:30 (Italian time), almost two hours later than the 3rd July flare peak (radiating 105 × 10^−22^ W m^−2^ Hz^−1^).

To show the effects of solar radio emissions at 2.6 GHz, the angular distribution of radiomobile measures around the direction of maximum BS cell power is presented. The angular distribution (reported as H_BEAM in the graphs) is defined as the horizontal angle formed by the direction of maximum power emission of a BS cell (cell azimuth) and the direction formed by the line joining the BS cell geographical position and the geographical position of the user at the time of the UL_SINR measure.

When the H_BEAM results zero, it means that the user is perfectly aligned with the direction of the BS cell maximum power emission (typically a good situation for the signal-to-noise ratio), whereas in the other cases, the user is less aligned to the maximum BS cell power, up to the extreme case (+ 180° or −180°), where the user exchanges only the very limited power that the BS cell receives or emits in retropropagation.

**Figure 6:**
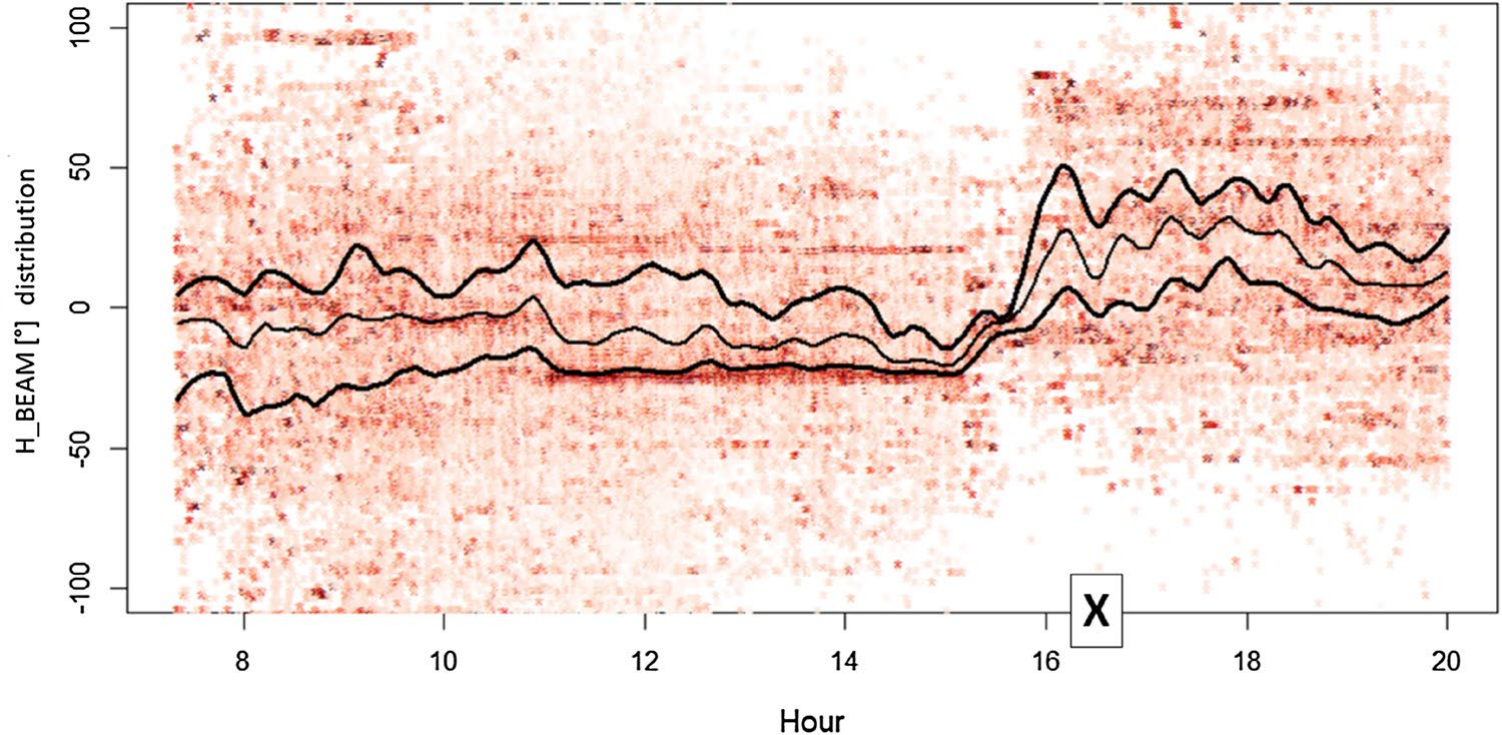
Angular distribution of measures around the direction of maximum power for BS cells Sun oriented during July 3rd. Lines identify the distribution median (the central line) and +/− standard deviation (color saturation reflects the density of cases). “X” indicates the flare peak in X-ray emission (local Italian time, UT + 2H). The loose of balance (distribution squeezing and bending) approaching “X” depends also on two contingency reasons: a sudden decrease of the number of measures for the Sun exposed BS cells and BS cells azimuths not perfectly distributed along all possible 0°–359° angles.

**Figure 7:**
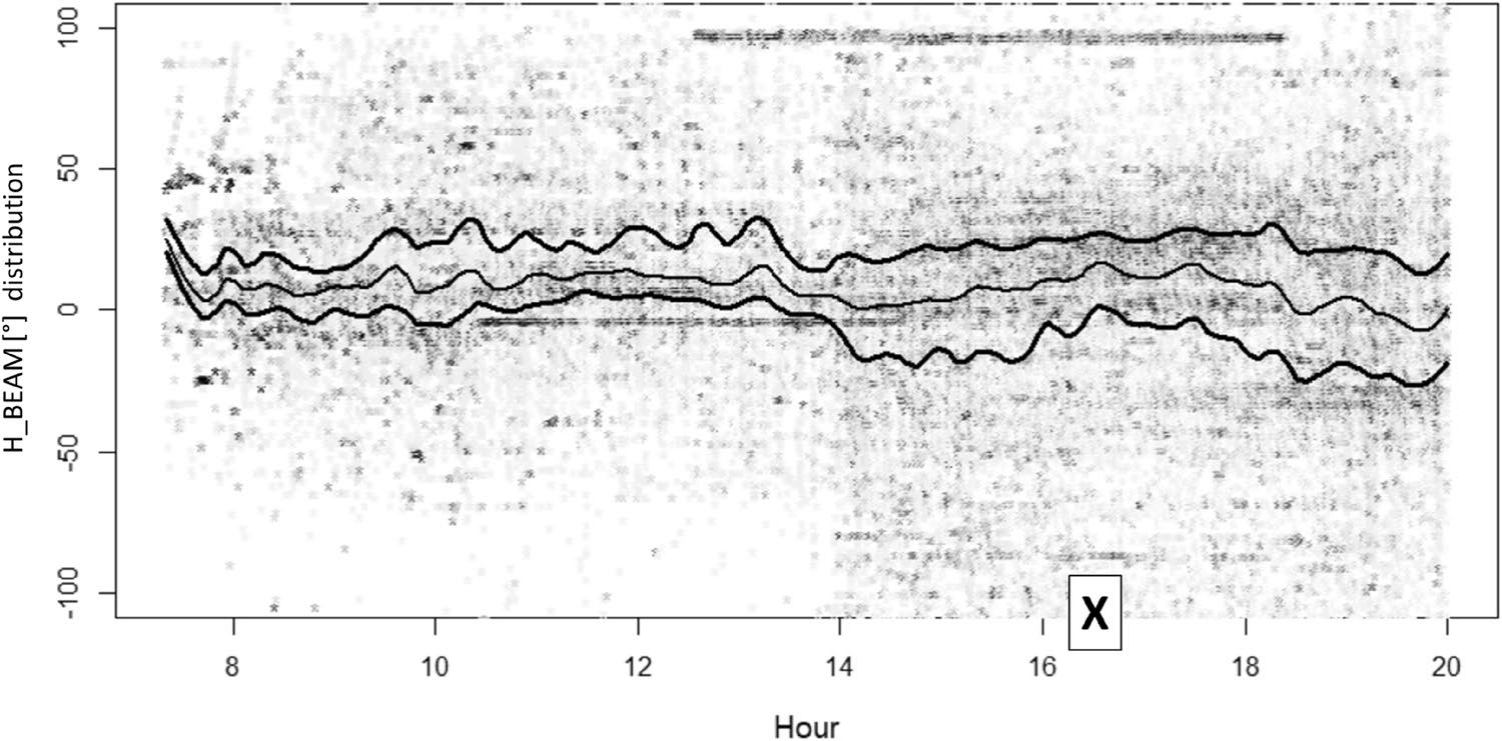
Angular distribution of measures around the direction of maximum power for BS cells not Sun oriented during July 3rd. Lines identify the distribution median (the central line) and +/− standard deviation deviation (color saturation reflects the density of cases). “X” indicates the flare peak in X-ray emission (local Italian time, UT + 2H).

**Figure 8:**
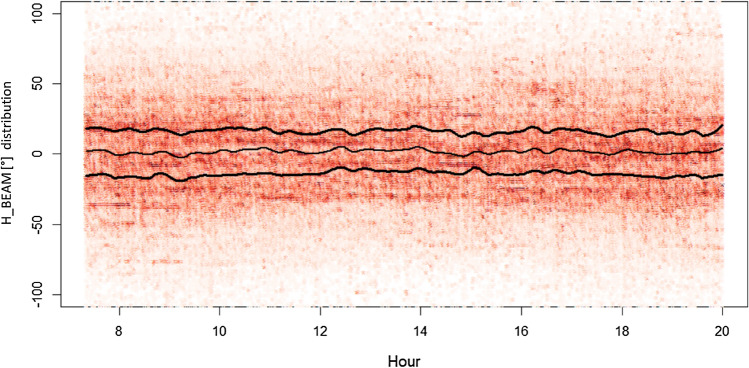
Angular distribution of measures around the direction of maximum power for Sun cells oriented during July 2nd. Lines identify the distribution median (the central line) and +/− standard deviation deviation (color saturation reflects the density of cases).

**Figure 9:**
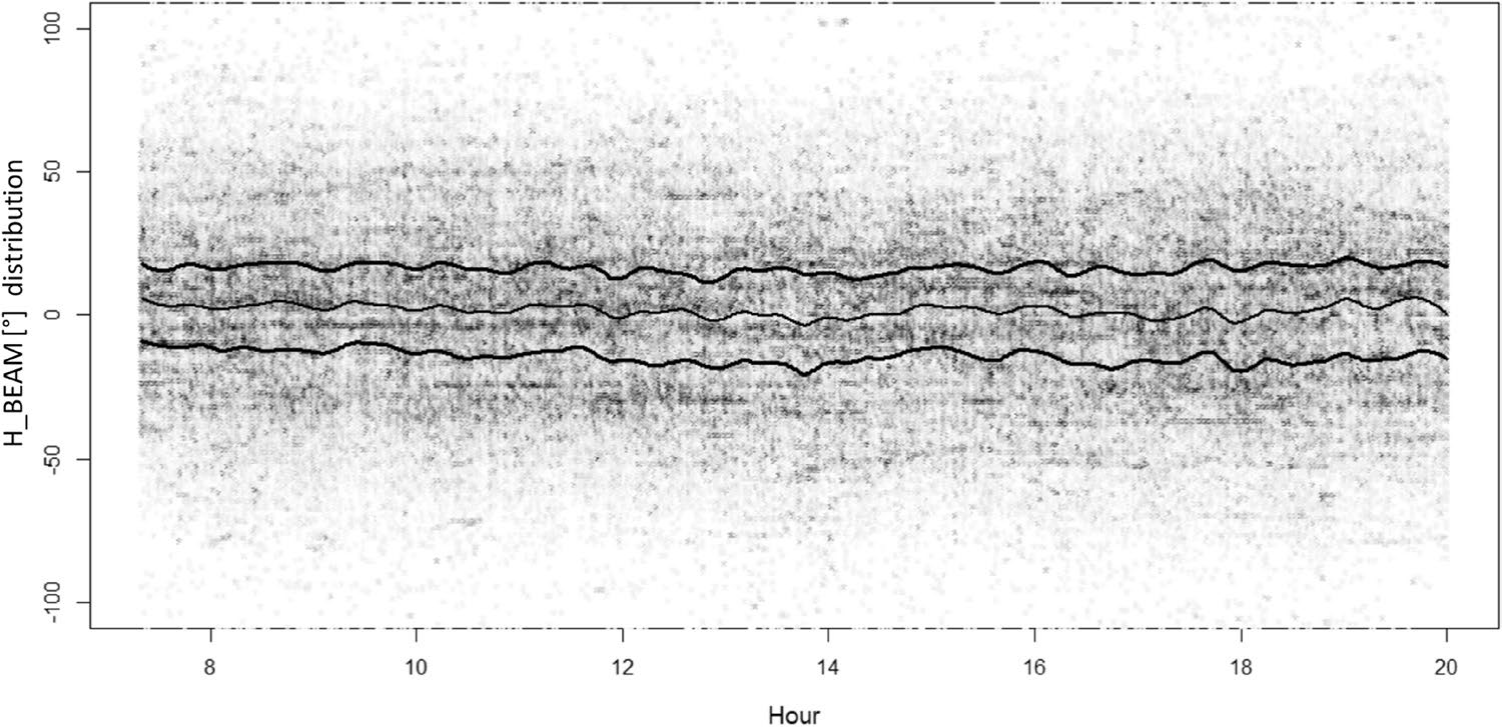
Angular distribution of measures around the direction of maximum power for cells not Sun oriented during July 2nd. Lines identify the distribution median (the central line) and +/− standard deviation deviation (color saturation reflects the density of cases).

The distortion of the H_BEAM angular distribution during the flare day (3rd of July 2021) afternoon is presented in Fig. 6 (BS cells Sun oriented, in red) and in Fig. 7 (BS cells not Sun oriented, in gray). To have an appropriate angular distribution benchmark during a day of quiet Sun, the same BS cells and the same angular distributions are calculated for the day before the 2nd of July 2021, again presenting results in red for Sun-oriented BS cells (see Fig. 8) and in gray for non-Sun-oriented BS cells (see Fig. 9).

It is not a surprise that, on both consecutive days, the H_BEAM distribution sees users concentrated almost around the maximum cell power direction (e.g., H_BEAM within +/− 30°), with queues sharply decreasing approaching the retropropagation cases (typically a bad situation for the signal-to-noise ratio).

## Methods

### Radiomobile data analysis

During the 3rd July Sun flare, the H_BEAM trend related to Sun-exposed BS cells was deeply affected. Specifically, during the two hours immediately preceding the flare (characterized by a greater background solar disturbance at 2.6 GHz), sun-exposed BS cells modified (squeezing, bending) their H_BEAM distribution (concentrating connections around the maximum cell power direction).

Afterwards, during the solar maximum X-ray emission time period (less background solar disturbance at 2.6 GHz) Sun-exposed BS cells relaxed their H_BEAM distribution (accepting more connections from the lateral direction of the cell). The reason behind this behaviour is the fact that BS cells having an azimuth close to the Sun azimuth receive solar noise from a direction aligned to the maximum antenna gain. If, at the same time, a mobile terminal tries to connect to the same Sun-exposed BS cell from a position largely not aligned to the maximum antenna gain direction (so at least laterally, with respect to cell azimuth), the signal sent by this lateral mobile phone encounters a lower (receiving) cell antenna gain. This badly influences the signal-to-noise ratio measured (UL_SINR) by the Sun-exposed BS cells and, as a consequence, badly influences the capability of the Sun-exposed BS cells to correctly decode lateral mobile phone requests of radio resources during the attach phase (when a mobile asks cells for RBs). In the case of unsuccessful attach, a cell mobile phone can try with a different cell, and if this new BS cell is not Sun exposed, the attempt can end with success. These types of mechanisms narrow the angular distribution for the Sun-exposed BS cells around the maximum antenna gain direction and, vice versa, can enlarge the angular distribution of the non-Sun-exposed cells.

**Figure 10:**
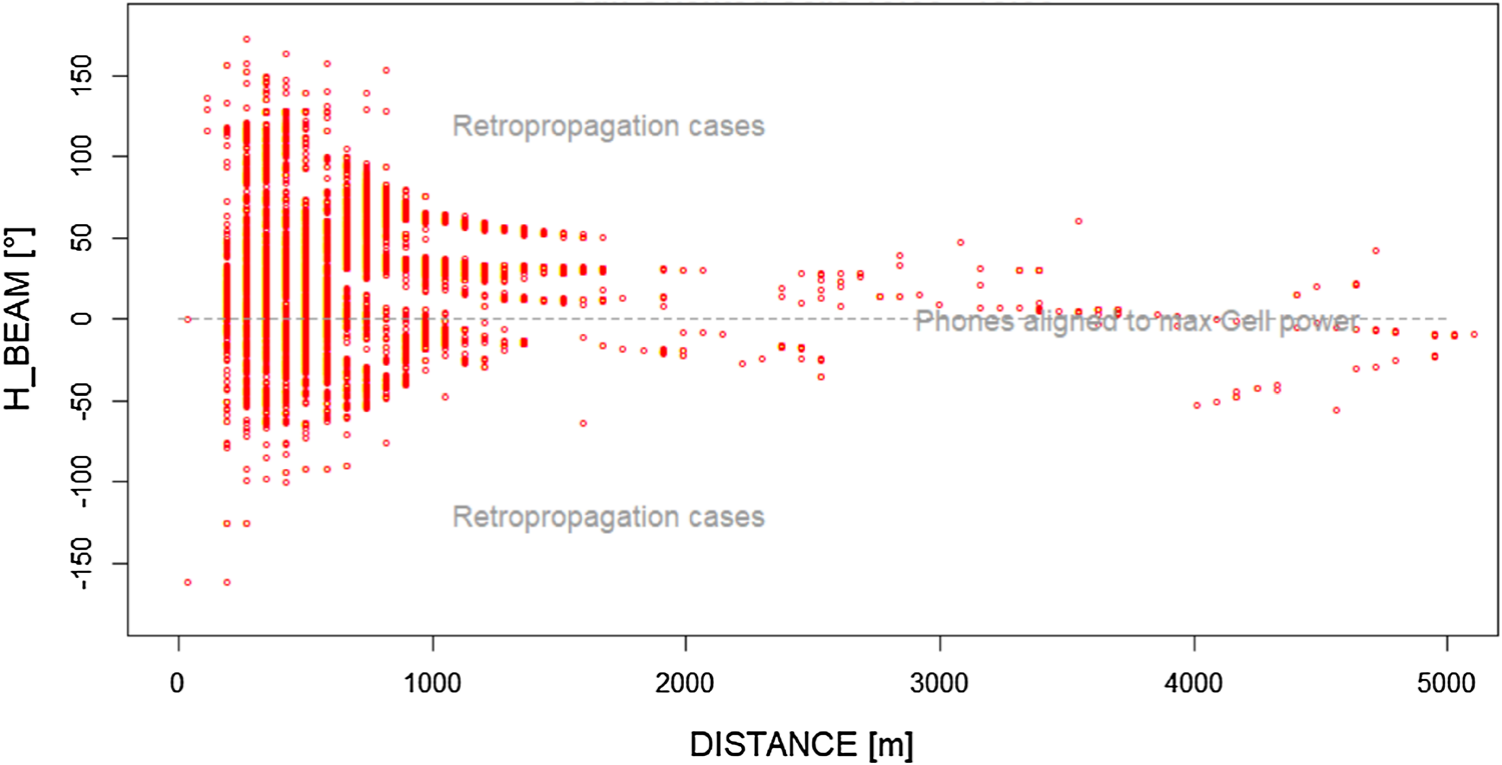
Angular distribution (H_BEAM) as a function of the electromagnetic distance of users from their serving BS cell, in the case of Sun-oriented BS cells during the two hours immediately before the 3rd July solar flare. The queues of the distribution (e.g., over + 100° and under −100°) are less populated with respect to the non-Sun-exposed BS cell equivalent queues (Fig. 11).

**Figure 11:**
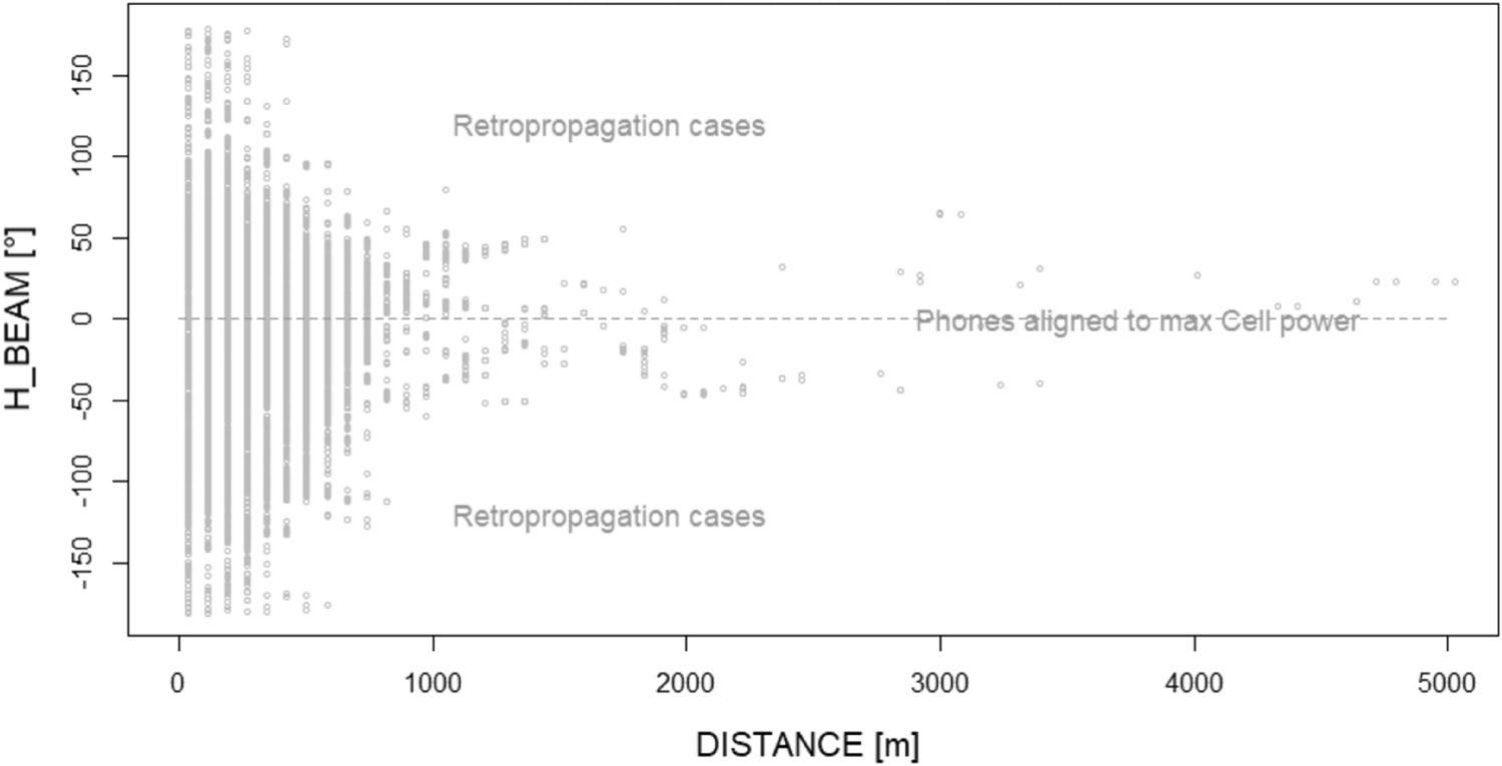
Angular distribution (H_BEAM) as a function of the electromagnetic distance of users from their serving BS cell, in the case of non-Sun-oriented BS cells during the two hours immediately before the 3rd July solar flare. The queues of the distribution (e.g., over + 100° and under −100°) are more populated with respect to the Sun-exposed BS cell equivalent queues (Fig. 10).

This effect could be graphically visible in Figs. 10 and 11, related to the two hours immediately before the flare start (14:00 to 16:00). These scatterplots show the differentiation of the network behaviour related to Sun-oriented and not Sun-oriented cells during a period of stronger solar disturbance.

Sun disturbance peaks (in radiomobile bands), in conjunction with the flexibility of the radiomobile procedures in BS cell selection, imbalance user allocation among available BS cells.

**Figure 12:**
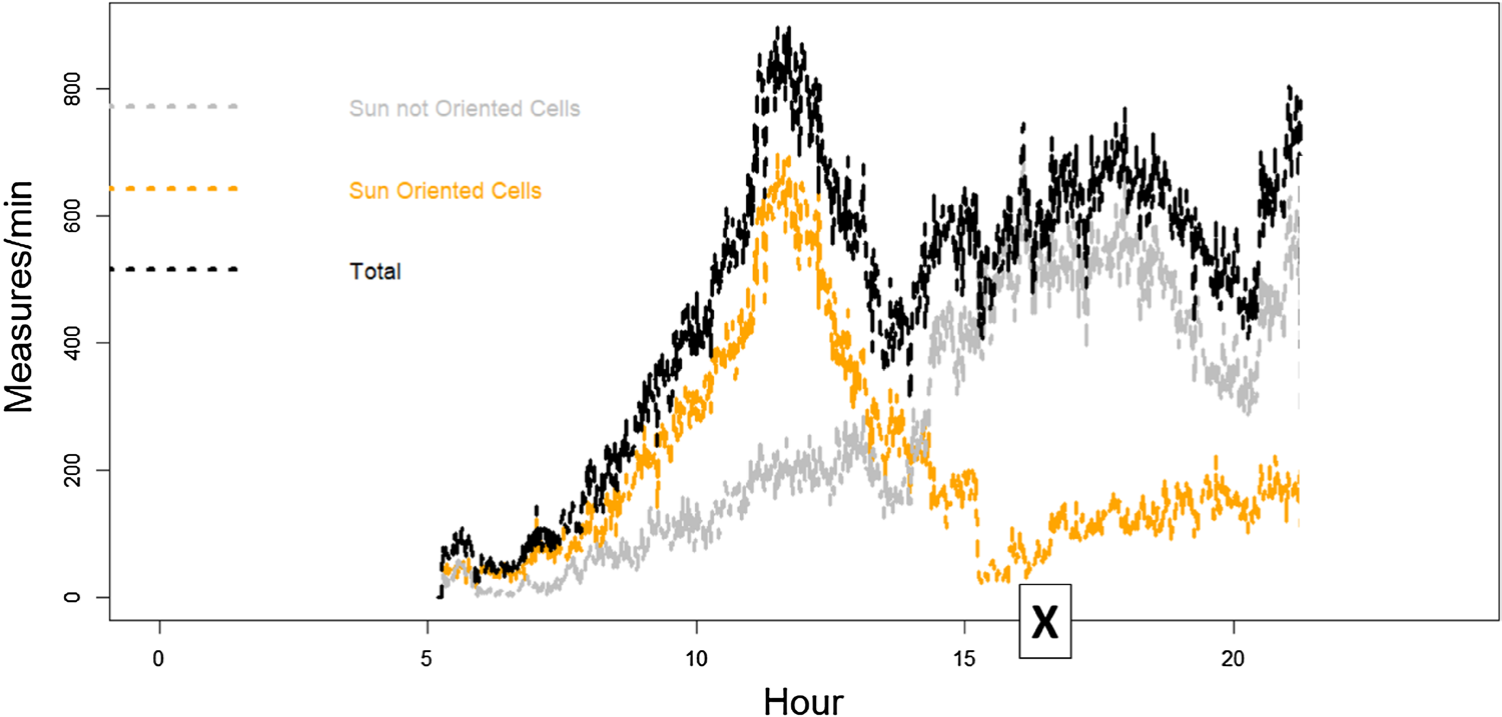
Example (Ferrara and Ravenna area) of the total number of connections (in black) during sunlight hours on the 3rd of July. The grey line identifies the part of the connection related to non-Sun-exposed BS cells, whereas the orange line identifies the part of the connection related to Sun-exposed BS cells. Trends are unbalanced. “X” indicates the flare peak in X-ray emission (local Italian time, UT + 2H).

**Figure 13:**
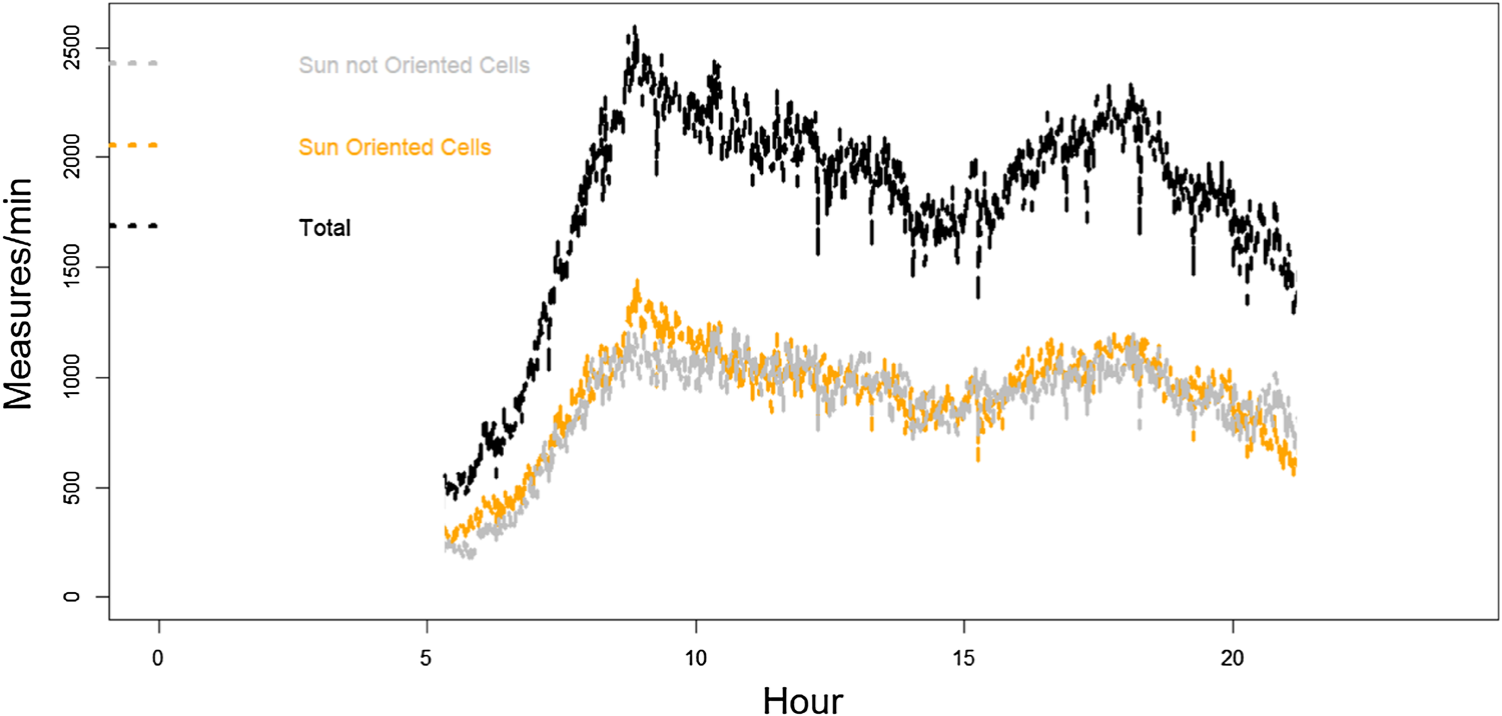
Example (Bologna area) of the total number of connections (in black) during the sunlight hours of the 2nd of July (no Sun flare). The gray line identifies the part of the connection related to non-Sun-exposed BS cells, whereas the orange line identifies the part of the connection related to Sun-exposed BS cells. Trends remain balanced all the day.

The network reaction to disturbances permits overcoming the imbalance, maintaining a stable total number of connections during the 3rd July solar disturbance period (Fig. 12). The day before, Sun quiet, shows a situation (Fig. 13) of perfect balancing among the same Sun-exposed and non-Sun-exposed BS cells.

The solar disturbance effect of depleting the number of users served by Sun-exposed BS cells and, on the other hand, improving the number of users served by non-Sun-exposed BS cells can be further analysed by defining the angular opening (around the BS cell azimuth) as the “H_BEAM Delta Correlation”, which includes 68% (a standard deviation) of the users served by that cell. When the possibility of serving users far from the main power emission is weakened by solar noise, the “H_BEAM Delta” tends to decrease (less lateral to retropropagation users). Conversely, when a cell also serves users laterally/retropropagation positioned, the “H_BEAM Delta” improves.

**Figure 14:**
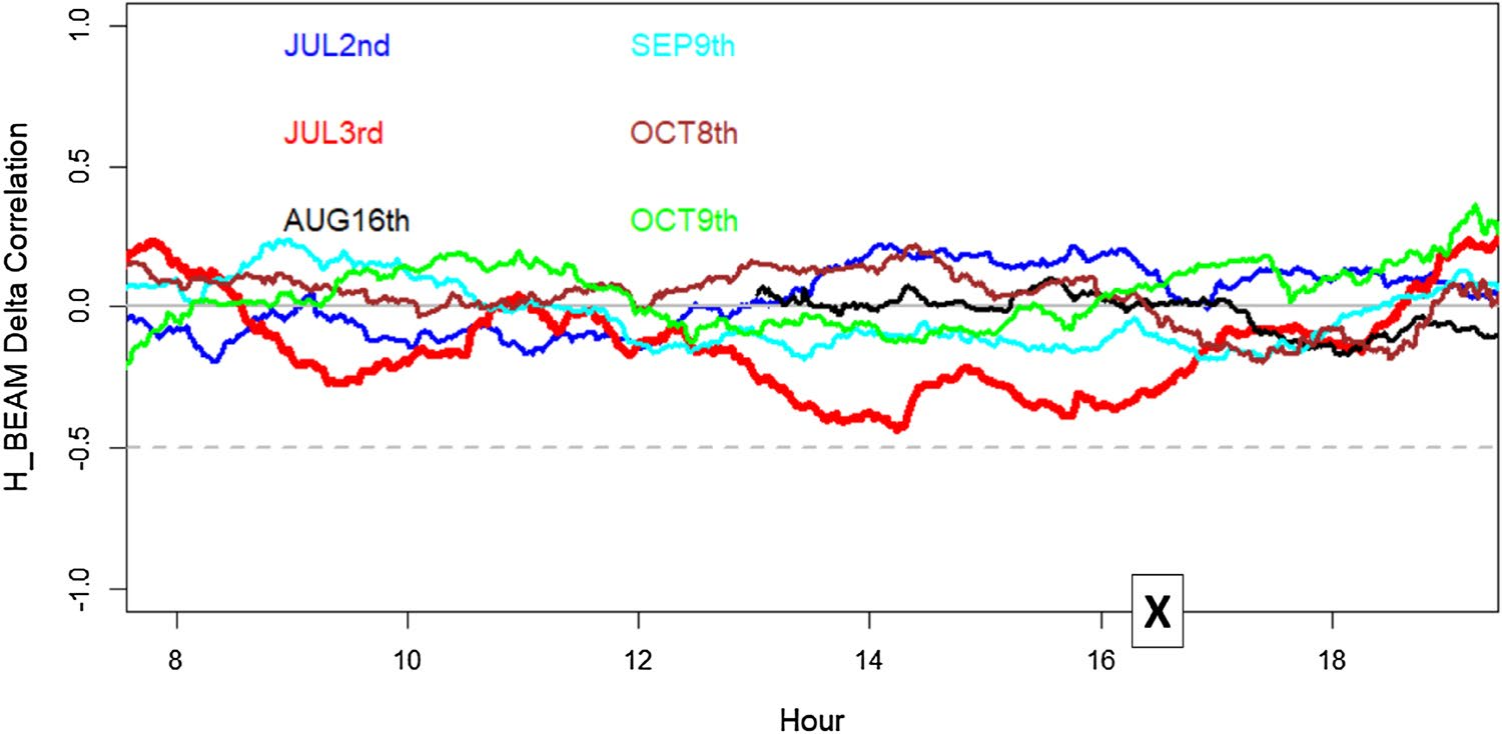
Correlation between the angular opening of BS cells (in relation to cell azimuth) including 68% of users (standard deviation of the distribution) related to Sun exposed BS cells and the angular opening of BS cells (in relation to cell azimuth) of not Sun exposed BS cells, using a sliding window of 180 min (at each minute the correlation value is attributed to the centre of the 180 min period), between sunrise and sunset. The “X” indicates the flare peak in X-ray emission (local Italian time, UT + 2H) related to the 3rd of July case.

Therefore, looking at the correlation between the “H_BEAM Delta” related to Sun-exposed BS cells and the “H_BEAM Delta” of non-Sun-exposed BS cells in general, we should not expect to see an anti-correlation because in a wide geographical area, the statistical behaviour of BS cells is expected to remain quite similar. In Fig. 14, the aforementioned “H_BEAM Delta correlation” is presented in relation to 6 different days, one of which is the 3rd July trend (red line), during which solar noise was much higher (flare peak at 16:29 local Italian time, UT + 2H). Along the other 5 days, Sun was less active (no flare with class comparable to that observed July 3rd). It is noticeable that the 3rd July “H_BEAM Delta correlation” trend becomes more negative (red line) during the hours preceding the flare. In that specific period, the network reacted to solar disturbance at 2.6 GHz assigning part of the radio resources to less disturbed BS cells (not Sun oriented), with the Sun oriented cells partially blinded by solar noise. This (temporary) lack of choice in selecting the best serving BS cell inevitably scaled down the overall service quality.

**Figure 15:**
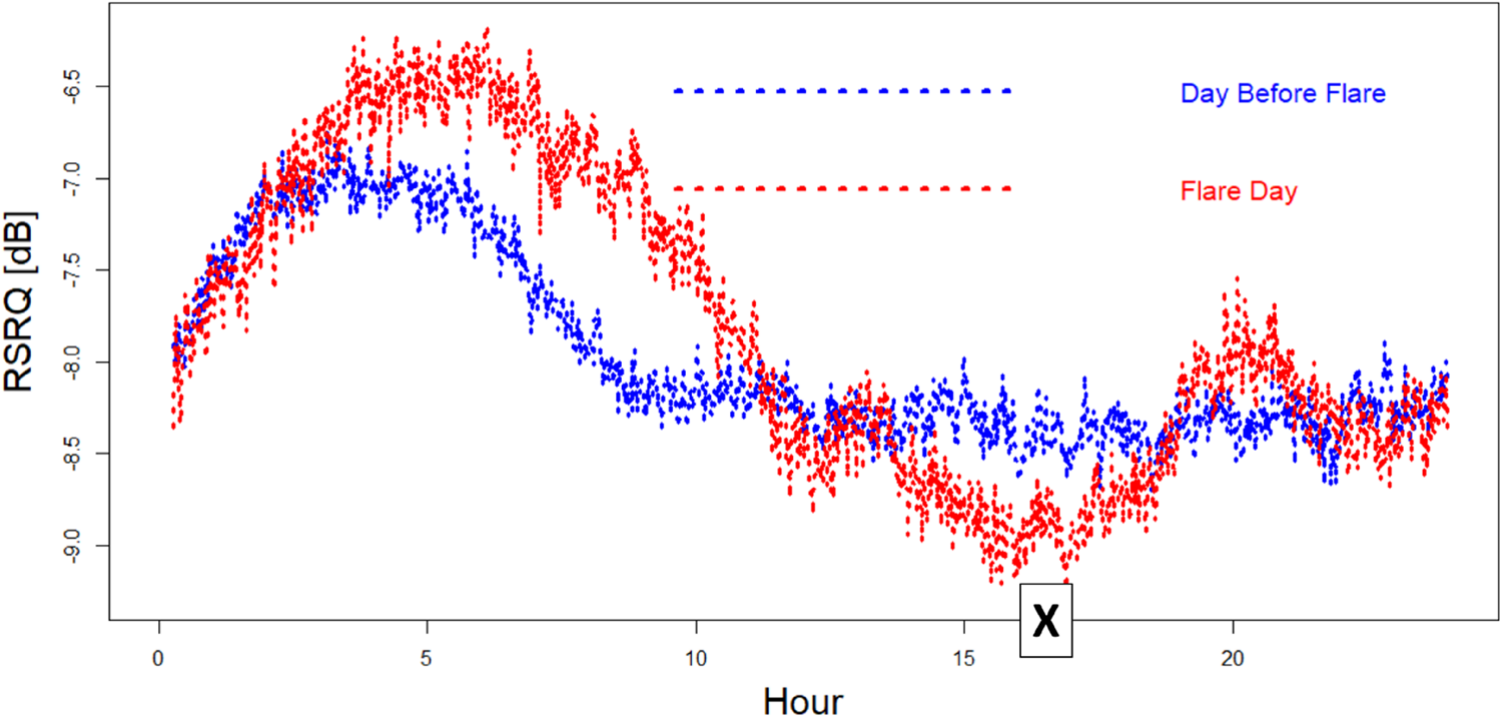
Comparison of RSRQ (received signal reference quality) measured by mobile terminals during July 2nd (blue line) and July 3rd (red line). A quality drop is visible for the hours close to the Sun flare. “X” indicates the flare peak in X-ray emission (local Italian time, UT + 2H).

The drawback of solar noise on quality is visible also analysing the LTE quality measures (RSRQ Reference Signal Received Quality) produced by the mobile terminals during their connections. Channel quality at 2.6 GHz could be affected (RSRQ measures the Downlink channel) by the fact that the best BS cell/channel assignment during stronger solar disturbance was limited (Sun-exposed BS cells were partially blinded to solar noise). This effect is visible in Fig. 15, where the RSRQ trend of July 3rd (disturbed by Sun) is compared with the RSRQ trend of July 2nd (more quiet Sun).

The hours with “H_BEAM Delta” anti-correlated are also characterized by a drop in mobile terminals received quality (RSRQ), further confirming the effects on radiomobile of (big) solar flares.

**Figure 16:**
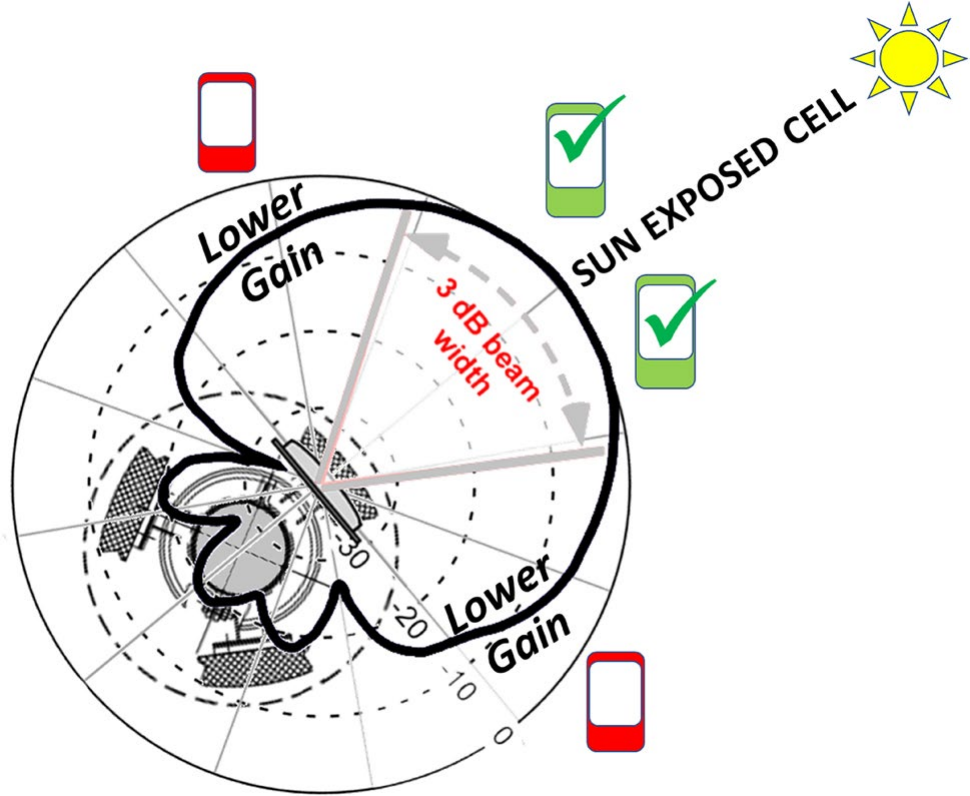
Scheme of a Sun-exposed BS Cell, where the connected phones laterally located (in red) experience a lower gain (thick black line) of the signal sent (Up Link) to the antenna. Conversely, the disturbing noise generated by the active Sun, which is more aligned to the cell azimuth, experiences the maximum receiving antenna gain. This situation unbalances the signal-to-noise ratio for the up link direction.

In term of noise power 1 s.f.u. (extremely low Sun noise) could inject in LTE BS cells a very low power, on average −161 dBm (assuming the UE average UpLink use of the 15 MHz band in the investigated areas and the average effective area of an ideal isotropic radiator at 2.6 GHz) solar disturbance remains almost invisible to radio-mobile (−130dBm being the lower limit for current 4G LTE connection in absence of interference) until three conditions are reached (see Fig. 16). Solar flux increases to much higher values (e.g., > 100 s.f.u.), solar disturbance enters the receiving BS cell from a direction corresponding to high antenna gain (close to cell azimuth) and user signal enters in the same receiving antenna from a direction corresponding to a low antenna gain (far from BS cell azimuth).

For example, with 300 s.f.u. (Sun active) average solar disturbing power grows at −137dBm. The 7 dB difference (in respect of −130dBm lower limit for current 4G LTE connection) allows the RAN to overcome the solar noise problem when both signals, the user signal and the noise, share the same direction (and thus the same antenna gain). If solar noise is less attenuated with respect to the user signal, the gap could be reduced up to a threshold that makes UL_SINR insufficiently robust (other sources of noise could also be present) for the connection. Obviously, exceptional events (solar noise emissions much higher than 300 s.f.u) could also unbalance the UL_SINR when solar noise and user signals share similar directions.
